# Supplementary material for: A CMOS-compatible oscillation-based VO2 Ising machine solver
Source: Nat Commun. 2024 Apr 18;15:3334. doi: 10.1038/s41467-024-47642-5 (PMC11026484; doi:10.1038/s41467-024-47642-5)
Supplement: Supplementary file 1 — Supplementary Information [file 41467_2024_47642_MOESM1_ESM.pdf]

Supplementary Information

VO_2 crossbar fabrication process

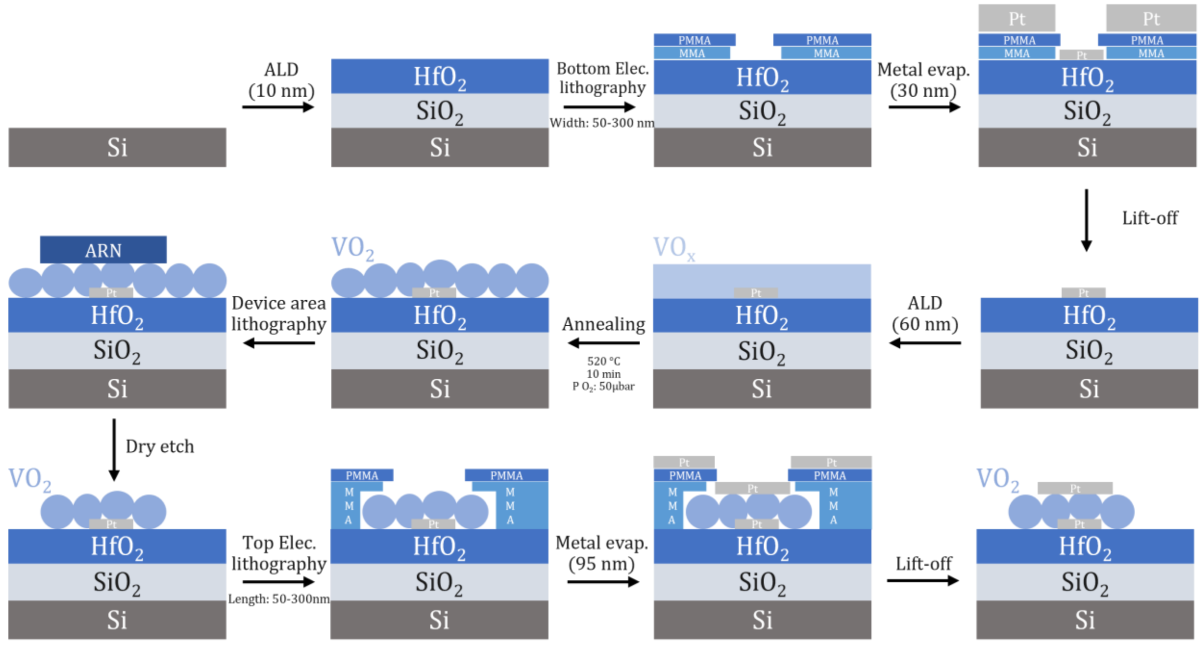

Figure S1. Fabrication process of VO_2 crossbar devices.

Figure S1 illustrates the fabrication process of the VO_2 crossbar devices. A thin layer (10 nm) of hafnium oxide (HfO_2) is deposited by atomic layer deposition (ALD) on a Silicon (Si) wafer (500 nm) with native silicon dioxide (SiO_2) (~2 nm). The bottom Platinum (Pt) electrodes are defined through e-beam lithography and deposited by metal evaporation (30 nm). Following the lift-off process, the amorphous vanadium oxide film is deposited by ALD (60 nm) with the Tetrakis[ethylmethylamino] vanadium (TEMAV) reaction at 150 °C, using Argon as the inert carrier gas and water as the oxidation agent. The wafer is annealed at 520 °C for 10 minutes under a constant oxygen partial pressure of 50 μbar to stabilize the film in the VO_2 state – see ref[1] for more details. A lithography process followed by a sulfur hexafluoride (SF6) plasma-based dry etch defines the device area.

The top Pt electrodes (95 nm) are subsequently deposited using the same method as the bottom electrodes.

Temperature dependent characteristics

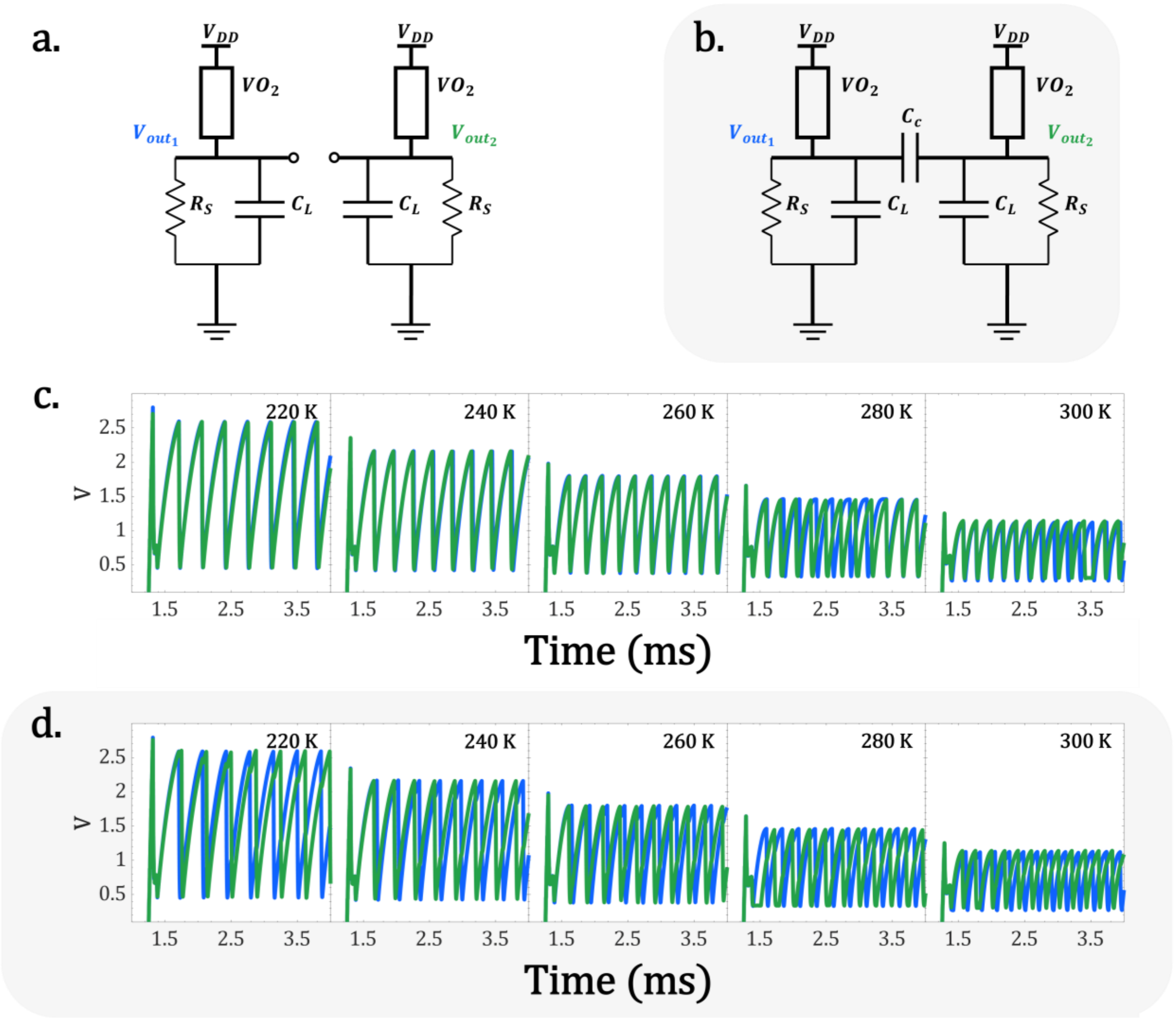

Figure S2. Temperature-dependent VO_2 oscillations. Two **a** uncoupled and **b** coupled VO_2-based oscillators with corresponding oscillating characteristics in **c** and **d.** The oscillation amplitude ($V = V_{DD}$-V_{out}) measured at the device terminals decreases with increasing temperature (active area: 100 × 50 × 60 nm^3).

Table S1. Values of the circuit parameters employed to conduct the temperature dependent measurements in Figure S2.

**Temperature** (K)	V_{DD} (V)	R_S (kΩ)	C_L (nF)	C_c (nF)	V_{TL} (V)	V_{TH} (V)	$f_{natural}$ (kHz)	$f_{coupled}$ (kHz)	**N. cycles to stable state when coupled**
**220**	5.50	40	10.0	0.68	0.46	2.60	2.90	2.75	5
**240**	5.50	40	10.0	0.68	0.45	2.15	3.35	3.20	5
**260**	5.75	40	10.0	0.68	0.40	1.80	4.05	3.90	7
**280**	5.85	40	10.0	0.68	0.34	1.44	4.40	4.07	3
**300**	4.50	30	10.0	0.68	0.29	1.12	4.90	4.75	5
**320**	7.00	30	10.0	—	—	—	—	—	—

Figure S2c-d presents the oscillating characteristics of two uncoupled VO_2-based oscillators in Figure S2a and coupled in Figure S2b. The oscillation amplitude ($V = V_{DD}-V_{out}$) measured at the device terminals decreases progressively with increasing temperature. This reduction is attributed to the lower power required to reach the material's switching points (see Figure S3d) when the ambient temperature is higher.

This trend is further illustrated by the reducing threshold voltages (V_{TL} and V_{TH}) reported in Table S1. The number of cycles required to reach the expected out-of-phase relationship, when the devices are coupled, remains fairly stable regardless of the ambient temperature.

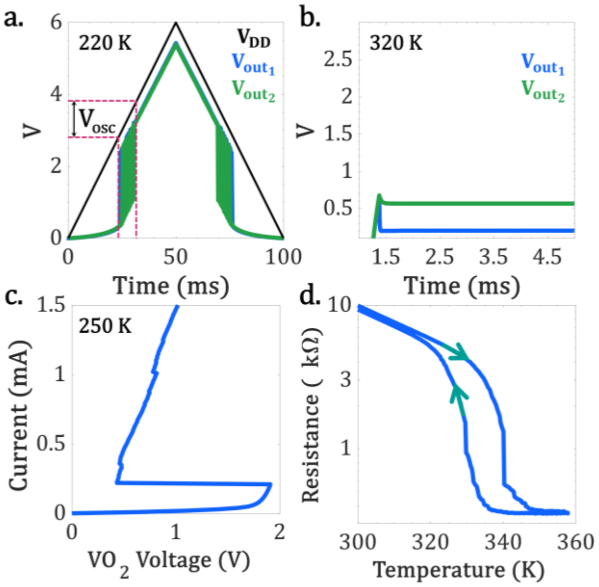

Figure S3. Additional VO_2 devices characteristics. a Output signals of two VO_2 devices connected to a series resistance (R_s = 10 kΩ) biased with a voltage ramp ranging from 0 V to 6 V. **b** Experimental waveforms of two uncoupled VO_2 oscillators at 320 K (active area: 100 × 50 × 60 nm^3, R_s = 5 kΩ, C_L = 10.0 nF, V_{DD} = 875 mV). **c** I-V (current-driven) graph of a VO_2 device at 250 K. (active area: 100 × 50 × 60 nm^3). **d** Resistance-temperature (R-T) characteristics of a VO_2 device (active area: 100 × 50 × 60 nm^3, R_s = 40 kΩ).

In Figure S3a, the voltage at the devices' outputs at 220 K, connected to a low series resistance (10 kΩ), are shown as the supply voltage is ramped. Oscillation occurs only within a specific voltage range (V_{osc}). With an increase in ambient temperature, this range becomes smaller, and the variability among VO_2 devices[1] makes it challenging to find a

supply voltage that aligns within V_{osc} for all devices simultaneously. This situation is represented in Figure S3b, where, at 320 K with the same V_{DD}, one device remains in a high (insulator) resistive state while the other remains in a low (metallic) resistive state. In this case, the absence of an oscillating pattern prevents successful device coupling. This motivates our decision to operate at low temperature (220 K) to ensure a broader range of viable operation modes under large biases (see Figure S3c).

Freeze-out effect

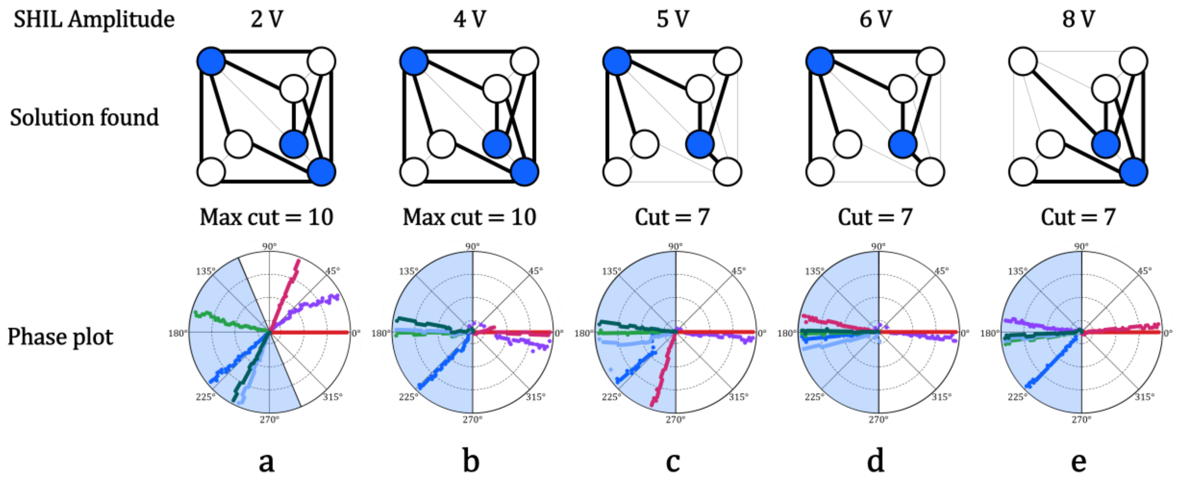

Figure S4. Freeze-out effect. Experimental phase relationships of coupled relaxation VO_2 oscillators, involving 8 nodes to solve the Max cut problem of a Mobius ladder. The SHIL signal amplitude is increased by steps – **a** 2 V, **b** 4 V, **c** 5 V, **d** 6V, and **e** 8 V – to demonstrate the 'freeze-out' effect when the amplitude is too high, trapping the system in a local minimum and preventing it from reaching the ground state.

Figure S4 illustrates the outcomes of the Max-cut problem mapped onto a network of seven VO_2 oscillators. The amplitude of the SHIL signal is the only adjusted parameter, varying from 2 to 8 Volts. Within the experiment depicted in Figure S1a, the system convergences towards the optimal solution employing a 2V amplitude. It achieves proper

state discretization only when the voltage reaches at least 4V (see Figure S4b). Beyond this voltage threshold, the system becomes trapped in a suboptimal solution, resulting in the network's inability to find the max cut of the problem (see Figure S4c, d, and e). This is a typical example of the ‘freeze-out’ effect.

Importance of electrical parameters

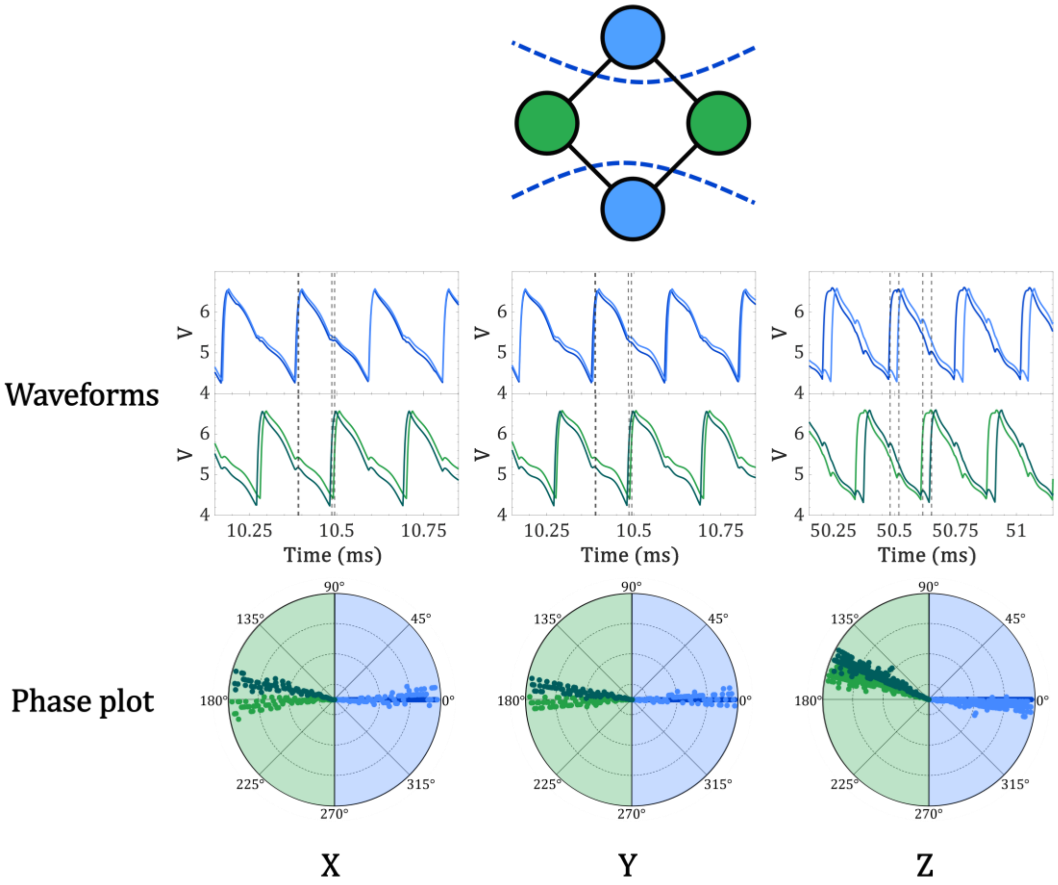

Figure S5. Graph coloring problem with varying circuit parameters. Experimental waveforms and phase relationships of coupled relaxation VO_2 oscillators, involving 4 nodes to solve the Max-cut problem. A low SHIL signal amplitude (X compared to Y) weakens the binarization of the states by increasing the cluster diameter, while excessive

increase in the coupling capacitance (Z compared to Y) leads to a steady state where the phases fail to maintain the expected 180° out-of-phase relationship.

Table S2. Values for the circuit parameters employed to solve the Max-cut problems in Figure S5.

Case	V_{DD}	R_S	C_L	C_c	C_{SHIL}	V_{SHIL}	*f*	Active area
**X**	7.0 V	30 kΩ	10.0 nF	**680 pF**	680 pF	**4.0 V**	4.75 kHz	300 ×1080 × 60 nm³
**Y**	7.0 V	30 kΩ	10.0 nF	**680 pF**	680 pF	**6.0 V**	4.75 kHz	300 ×1080 × 60 nm³
**Z**	7.0 V	30 kΩ	10.0 nF	**2.2 nF**	680 pF	**6.0 V**	4.75 kHz	300 ×1080 × 60 nm³

Figure S5 shows the experimental results of the Max-cut problem for a graph involving four oscillators coupled in a square configuration. The values of the parameters used for the three different experiments are reported in Table S2. By comparing the waveforms and the phase plots in Figure S5 X and Y, it becomes apparent that a high amplitude in the injected signal leads to a reduction in the cluster diameter within each subset or state. When the coupling capacitance C_c is excessively large, as illustrated in Figure S5 Z where C_c (2.2 nF) exceeds 10% of C_L (10 nF), it becomes more difficult for the system to reach the ground state and ignore some of the constraints imposed by the connection between outputs. The average phase difference between the binarized states lowers from 171° (Figure S5 Y) to 155° (Figure S5 Z), evidencing the importance of carefully adjusting the parameters for each specific graph to maximize performance.[2]

Values of the circuit parameters

Table S3. Values of the circuit parameters employed to solve the Graph coloring problems in Figure 3.

Graph	V_{DD} (V)	R_S (kΩ)	C_L (nF)	C_c (nF)	Active area (nm^3)
**Northern Europe**	5.5	40	10.0	2.2	100 × 50 × 60
**Central Europe**	9.0	40	10.0	0.68	300 × 300 × 60
**East Asia**	7.0	30	10.0	2.2	300 × 80 × 60
**South America**	5.5	42	11.5	1.0	100 × 50 × 60

Table S4. Values of the circuit parameters employed to solve the Max-cut problems in Figure 5.

Graph	V_{DD} (V)	R_S (kΩ)	C_L (nF)	C_c (nF)	C_{SHIL} (nF)	V_{SHIL} (V)	*f* (kHz)	Active area (nm^3)
**A**	7.0	30	10.0	0.68	0.68	6.0	4.75	300 × 80 × 60
**B**	9.0	40	10.0	0.68	0.47	2.2	4.90	300 × 300 × 60
**C**	9.0	40	10.0	1.0	0.68	6.0	4.15	300 × 300 × 60
**D**	7.0	50	10.0	0.68	0.47	4.0	3.05	200 × 200 × 60
**E**	6.0	43	10.3	1.0	1.5	2.0	2.04	150 × 150 × 60
**F**	7.25	44	10.0	0.68	0.27	4.0	2.92	250 × 250 × 60
**G**	5.5	44	10.3	1.0	1.5	2.0	1.69	100 × 50 × 60

Table S5. Values of the circuit parameters employed to solve the MAX-3SAT problems in Figure 8.

Graph	V_{DD} (V)	R_S (kΩ)	C_L (nF)	C_c (nF)	$C_{2\text{-}HIL}$ (nF)	$C_{1\text{-}HIL}$ (nF)	$V_{2\text{-}HIL}$ (V)	V_h (V)	f (kHz)	Active area (nm^3)
$\mathcal{F}_1$	9.0	45	10.0	0.68	0.47	0.27	6.0	4.0	3.10	300 × 300× 60
$\mathcal{F}_2$	6.75	48	10.0	1.0	0.47	0.27	9.0	4.0	2.47	150 × 150 × 60
$\mathcal{F}_3$	6.75	46	10.0	1.0	0.27	0.47	6.0	8.0	2.33	150 × 150 × 60

References

1. Maher, O. *et al.* Highly Reproducible and CMOS-compatible VO2-based Oscillators for Brain-inspired Computing. https://doi.org/10.48550/arXiv.2403.02822

2. Zhang, Y. *et al.* Oscillator-Network-Based Ising Machine. *Micromachines (Basel)* **13**, 1016 (2022).
